# Opportunities and Challenges for Designing in Connected Health: Insights From an Expert Workshop

**DOI:** 10.2196/72446

**Published:** 2025-10-31

**Authors:** Shweta Premanandan, Awais Ahmad, Alex Jaranka, Ece Üreten, Eunji Lee, Jennifer Gross, Åsa Cajander, Sofia Ouhbi, Marta Lárusdóttir, Owen Eriksson, Sten-Erik Öhlund

**Affiliations:** 1 Division of Computing Science Department of Information Technology Uppsala University Uppsala Sweden; 2 Vi3 Department of Information Technology Uppsala University Uppsala Sweden; 3 Department of Neurobiology, Health Sciences and Society Karolinska University Stockholm Sweden; 4 Control and Simulation Group Faculty of Aerospace Engineering Delft University of Technology Delft The Netherlands; 5 Biomedical Signals and Systems Chalmers University of Technology Gothenburg Sweden; 6 Department of Computer Science Reykjavik University Reykjavik Iceland; 7 Department of Informatics and Media Uppsala University Uppsala Sweden

**Keywords:** connected health, participatory expert workshop, eHealth, digital health, medical informatics, interoperability, digital health systems regulations, sustainability in digital health, designing for connected health, digital infrastructures

## Abstract

Health care increasingly depends on information and communication technology. This offers both opportunities and challenges when designing connected health systems. While individual studies examined particular cases, there is a limited synthesis of insights across projects. The objective of this paper is to explore these opportunities and challenges by examining 6 diverse connected health projects and synthesizing lessons from an expert workshop. To achieve this, we conducted a full-day workshop that brought together 6 connected health projects. The workshop used an iterative and participatory process which included paper submissions and presentations and facilitated discussions, and a gallery walk to enable cross-case comparison and collaborative reflection. Thematic analysis of workshop outputs was then used to synthesize key opportunities and challenges in designing connected health systems. The 6 projects represented a variety of design methods and approaches to connected health, and their discussion surfaced both opportunities and challenges in this domain. Key opportunities include improving data integration and usability, enhancing collaboration across stakeholders, using a user-centered and iterative design process, addressing complexity in sociotechnical systems, sustainability, and adopting digital infrastructures for seamless communication. Participants also identified important challenges, namely exchange of information, interoperability, and communication; ethical considerations, rules, and regulations; understanding design, evaluation, and standards; actionable data, reliability, quality, and trust in data; and stakeholder involvement. The contribution of this paper lies in the synthesis of insights across multiple projects and perspectives to provide practical guidance for researchers, designers, and policymakers. By highlighting opportunities and challenges in designing connected health systems, the findings emphasize the importance of patient-centered, sustainable, and collaborative design approaches while also pointing to the need to address persistent barriers. Advancing connected health will require adopting iterative and inclusive design processes that prioritize patient-centeredness, sustainability, and collaboration across health care systems.

## Introduction

### Overview

The rapid digitization of health care resulted in connected health systems, created opportunities to enhance patient care and optimize workflows, and fostered interdisciplinary collaboration [1]. However, these advancements also bring challenges related to user engagement, workflow integration, privacy, and regulatory compliance [2,3]. Addressing these challenges requires tailored design methods that align with the unique demands of connected health.

Connected health is an emerging field that integrates smart technologies into health care to improve patient outcomes and reduce costs [4]. It involves the logical linking of health-related elements and conditions [5] and has seen steady growth, particularly in technology-driven solutions for managing medical conditions [6]. To fully realize its potential, the aforementioned challenges must be addressed [6].

It requires careful consideration of needs from the stakeholders: patients, health care providers, and regulatory bodies. Persistent barriers include ensuring user engagement, integrating systems into clinical workflows, and navigating complex regulatory frameworks [7]. Additionally, achieving sustainability remains a key challenge [7]. This calls for interdisciplinary approaches to bridge technological capabilities with real-world health care needs.

This paper builds on the insights from an expert workshop focused on design methods for developing connected health systems. The workshop examined how design can serve as a critical enabler in overcoming technical and organizational barriers while ensuring systems meet diverse user and stakeholder needs. Through presentations, gallery walks, and collaborative discussions, participants explored 6 connected health projects: remote patient monitoring in primary care; intensive care unit (ICU) handover support with ecological interface design (EID); service process learning cycle for health care workflows; sustainable health system software co-design; the Swedish National Medication List for Interoperability; and KokemUX, a user-centered design for connected health.

### Why Design Matters in Connected Health

Connected health leverages information and communication technologies to improve health care quality and outcomes [8]. By integrating digital innovations into health care, connected health has the potential to transform health care systems, improving safety, quality, and efficiency. This is especially evident in cancer care, where patient-centric models supported by connected health interventions can significantly enhance the quality of life [9].

However, the increasing reliance on software-based health care solutions necessitates a deeper understanding of regulatory frameworks, particularly those governing Software-as-a-Medical Device [10]. Navigating these regulations is critical for fostering innovation while ensuring compliance and patient safety in connected health [10]. To develop effective, safe, and equitable health care interventions, it is essential to adopt user-centered, multidisciplinary standards in designing, developing, and implementing connected health solutions [9].

User-centered design (UCD) approaches play a crucial role in ensuring that connected health systems are usable and effective, particularly for older adults who are among the primary users of these technologies [11-13]. To integrate user needs throughout the rapid development process of connected health products, a structured 3-phase methodology has been proposed. This methodology includes use case construction, expert usability inspections, and user testing [12]. Despite these advancements, the business aspects of connected health remain underexplored [6]. By business aspects, we refer to the economic viability and sustainable business models of connected health technologies—including funding mechanisms, reimbursement schemes, cost-effectiveness, and organizational models that support scaling—rather than implying that financial considerations override patient health. This includes investigating how connected health initiatives can generate returns or create value in the health care system (eg, through cost savings, new revenue streams, or integration into care pathways) and how investments in patient health can align with financially sustainable structures [14-16]. Ethical considerations, such as balancing profit motives with equity and patient outcomes, are inherently linked to these business considerations and are acknowledged in our framing.

Addressing the challenges posed by an aging population and the increasing number of chronic patients requires optimizing data sensing, analyzing large datasets, and designing innovative care models that leverage technology platforms [17]. Additionally, UCD methods have proven valuable in addressing barriers to health care innovation [18].

Connected health offers significant opportunities for improving health care delivery, particularly when combined with big data analytics. These technologies enable innovative solutions that benefit both patients and health care providers [19]. However, the field also faces challenges, including regulatory compliance and risk mitigation [10,20]. The Internet of Health Things presents new opportunities for design research to address these challenges and shape the future of health care [21]. Key areas for design contribution include improving the safety, quality, and efficiency of health care services [21]. The successful implementation of connected health solutions has the potential to improve health care services and create global market opportunities [20].

The workshop discussed six different design methods in various connected health contexts:

1. **User-Centered Design:** It is an overarching design approach that focuses on creating IT applications tailored to users’ specific needs and preferences [22]. Under this umbrella, various approaches, such as participatory design, design thinking, positive design, and persuasive design, are included.
2. **Ecological Interface Design:** With its roots in cognitive work analysis, an ecological interface displays information within defined system constraints in a way that supports the user’s decision-making in complex sociotechnical systems and helps the user become an adaptive problem solver [23]. In connected health, EID can be used to design dashboards or monitoring displays that effectively integrate patient data, enabling health care providers to make informed decisions [24].
3. **Service Design:** Service design applies design thinking to improve services by focusing on interactions between users, technology, and processes [25]. In connected health, this approach ensures that care delivery is seamless, incorporating both digital tools and human interactions for a better patient experience.
4. **Design for Sustainability:** Designing for sustainability considers the functionality and purpose of the product and software over a time period, weighing the impacts on the different dimensions and scope levels [26]. One way this can be achieved is by examining the impact of software at different dimensions and levels [27]. There can be direct, indirect, and systemic impacts in social, technical, environmental, and economic dimensions from software [28]. A third aspect of software sustainability is how it is defined. Sustainability-in refers to the sustainability of the software itself. Sustainability-by means the software helps make another system more sustainable [29]. Connected health can include considerations such as resource-efficient systems that support health goals or sustainable designs that have minimal impact on the different dimensions.
5. **Infrastructural Design:** This method focuses on designing systems that integrate and support existing technological and organizational infrastructures. In connected health, infrastructural design ensures that new tools are compatible with existing health care systems, facilitating interoperability and scalability [30].
6. **KokemUX Design:** Derived from the Finnish word **kokemus** (experience), KokemUX design focuses on holistic user experiences by emphasizing emotional, social, and contextual dimensions. In connected health, it enhances patient engagement by addressing not only functional usability but also the emotional and experiential aspects of technology use [31].

### Viewpoint Focus and Guiding Question

This viewpoint builds on insights from an expert workshop, where 6 connected health projects were presented, each using different design methods. The full-day workshop included presentations followed by interactive activities such as gallery walks, discussions, and collaborative reflections. The expert workshop followed a structured, 3-stage iterative process, refining insights at each step. Discussions focused on key opportunities and challenges in connected health. By synthesizing these insights, the study highlights opportunities and challenges in designing connected health systems to complex health care environments. These findings provide valuable guidance for developing innovative and sustainable solutions that effectively meet the needs of patients, health care providers, and other stakeholders.

**Research Question:** What are the opportunities and challenges that arise when designing for connected health systems?

## Context and Approach (Expert Workshop as a Catalyst)

### Overview

This paper explored the opportunities and challenges in designing for connected health using an iterative and participatory approach. An expert workshop conducted in Uppsala, Sweden, on October 13, 2024, brought together researchers, practitioners, and design researchers from Sweden and other countries to discuss and refine insights from existing connected health projects. Participants voluntarily shared their work in a professional setting, contributing to a collaborative exchange of knowledge.

The agenda was planned collaboratively by the organizers, starting from the overall aims of the workshop, to explore diverse design methods in connected health, foster knowledge exchange across disciplines, and generate a joint scientific output. With these goals in mind, the organizers selected interactive formats, which are fishbowl discussions and a gallery walk, that would create space for sharing experiences, comparing approaches, and identifying common themes. The flow of the day was deliberately structured to begin with open discussions, move into more focused thematic exploration, and conclude with a wrap-up session. Preworkshop planning also involved circulating the workshop abstract and background information to participants in a shared folder so they could familiarize themselves with the scope and prepare to engage fully in the sessions.

Our positionality was informed by the multicultural and multicontextual nature of the research team. It brought together expertise in human-computer interaction, medical informatics, software engineering, and information systems, alongside the perspectives of researchers, designers, and physicians at different career stages, from PhD students and postdocs to professors. This diversity shaped how we approached the workshop and interpreted its outcomes. Our varied disciplinary backgrounds fostered sensitivity to both technical design considerations and clinical realities, while our international experiences encouraged us to reflect on how context shapes connected health practices. At the same time, we recognize that our positionalities—rooted in academic, design, and medical perspectives—may privilege certain framings of connected health (eg, usability, data integration, or workflow alignment) over others, such as policymaking. While this lens strengthened our ability to identify opportunities and challenges in design practices, we acknowledge that our interpretations may also carry implicit biases, such as overemphasizing technological feasibility, assuming access to digital infrastructures, or focusing on clinical workflows more than patient or policymaker perspectives.

### Participants and Procedures (Workshop Snapshot)

This workshop was conducted over the course of a full day, lasting a total of 7 hours. A call for participants was issued 4 months prior to the workshop, providing detailed information about the event and inviting submissions from prospective attendees. Participants were informed of the workshop’s purpose in advance, and consent was implied through voluntary registration and active participation. No identifiable information was collected, and workshop outputs, such as notes and collaborative documents, focused solely on methodological insights. No financial or material compensation was provided, as participation was voluntary and motivated by professional and academic interest.

The workshop was part of an international conference held in Uppsala. The call was disseminated through multiple channels, including the research group’s blog, international mailing lists, and the organizers’ professional networks. In addition, the organizers promoted the workshop on social media platforms—LinkedIn, Twitter, and Facebook—both through their own accounts and in relevant connected health and user experience design groups. Targeted email campaigns were also used to reach academics, health care professionals, and designers engaged in connected health. The call aimed to attract researchers, practitioners, and designers with an interest in practical design methods and interdisciplinary collaboration in the connected health domain.

**Figure 1:**
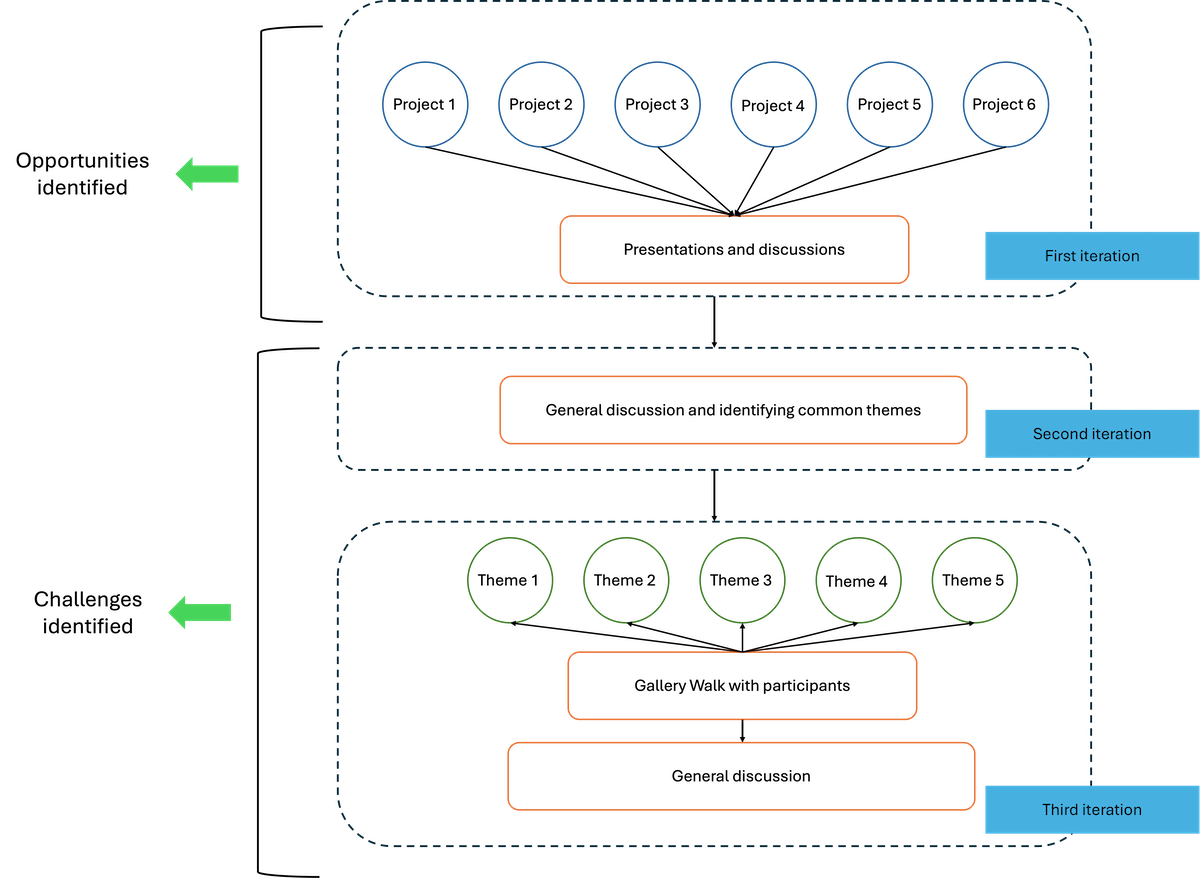
Overview of the method.

Interested participants were required to submit an abstract, following a specified template, outlining their connected health projects and describing the design methods or scenarios used. More information on the workshop is provided in the workshop paper [32]. A total of 8 participants applied and were selected to join the workshop; however, 1 participant later withdrew due to logistical challenges. Two of the workshop organizers also participated, and the total participants were 9. The participants brought diverse levels of experience in connected health, ranging from 2 to 20 years, enriching the discussions with a broad spectrum of perspectives and insights. The workshop process unfolded in 3 stages, each narrowing the focus and contributing unique insights to the final analysis. An overview of the method is illustrated in Figure 1. An overview of the participant information is provided in Table 1.

**Table 1:** Participant information.

Participants	Professional background	Experience in CH^a^ (in years)
P1	Family medicine and primary care	11-15
P2	Systems design engineering, human factors, and health informatics	11-15
P3	Health informatics and service design	11-15
P4	Computer science, software sustainability and quality, and health informatics	1-5
P5	User-centered design, human-computer interaction, agile software development, and health informatics	16-20
P6	Conceptual modeling, information systems, socioinstitutional ontologies, and health informatics	11-15
P7	Information systems, systems development, and health informatics	11-15
P8	Human-computer interaction, health informatics, and agile software development	16-20
P9	Software engineering, human-computer interaction, sustainability, and health informatics	11-15

^a^CH: connected health.

### First Iteration: Project Presentations and Discussions

Seven participants presented 6 connected health projects, providing a brief overview of their project’s objectives, methodologies, and key findings for 10 minutes. This was followed by discussions for 15 minutes on the individual project presentations. This phase allowed participants to familiarize themselves with each other’s work, highlighting the breadth of design practices and their applications in connected health. Opportunities were identified through these presentations and their discussions.

### Second Iteration: Thematic Discussions

**Figure 2:**
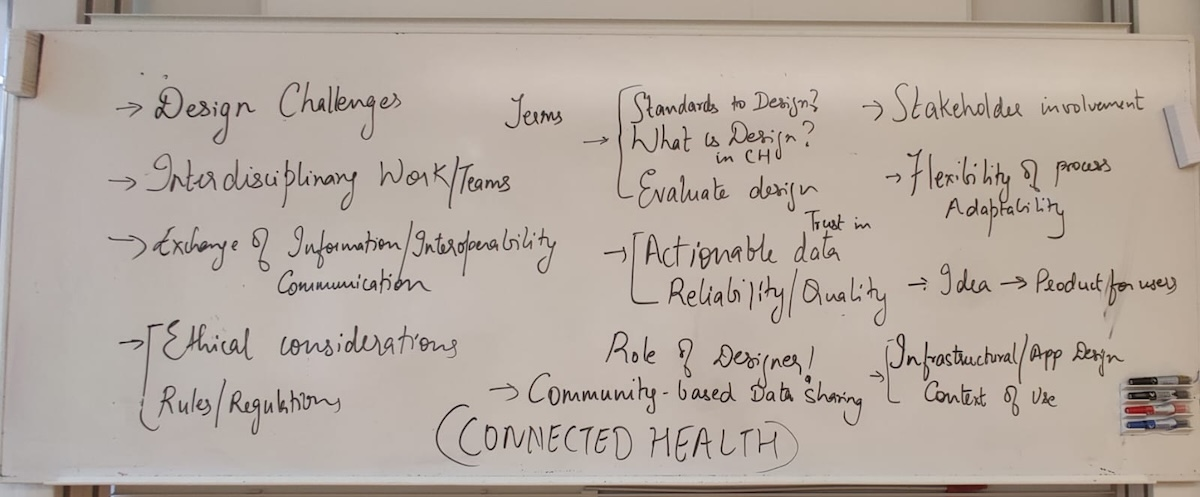
Brainstorming of themes after the first stage.

Following the presentations, participants engaged in a facilitated 2-hour discussion to collaboratively analyze and synthesize insights across the projects. This structured dialogue allowed for the identification of key crosscutting themes, representing shared challenges observed in the design and implementation of connected health technologies. These themes are shown in Figure 2. Through this process, five particularly significant themes emerged: (1) exchange of information, interoperability, and communication; (2) ethical consideration, rules, and regulations; (3) understanding design, evaluation, and standards; (4) actionable data, reliability, quality, and trust in data; and (5) stakeholder involvement. These were the basis for challenges when designing in connected health, and they were built on in the next phase.

### Third Iteration: Gallery Walk

**Figure 3:**
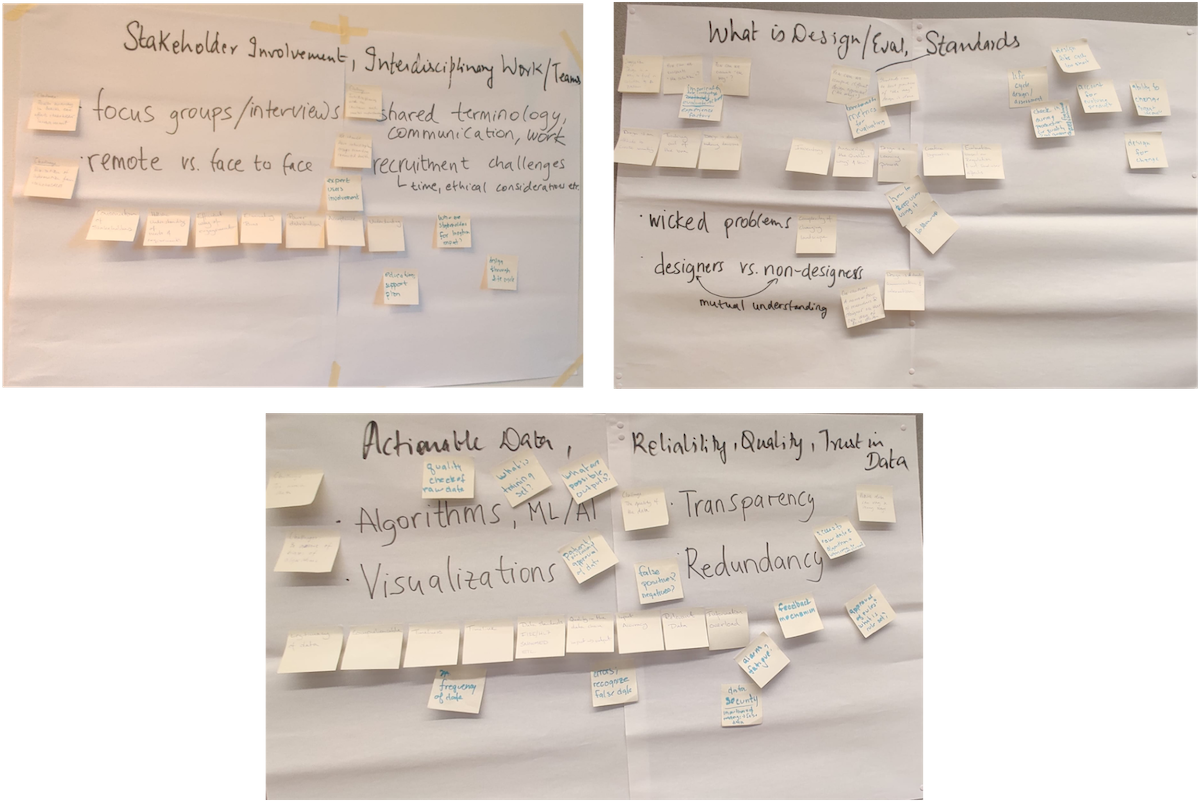
Examples of the Gallery Walk stations.

In this phase, the 5 themes identified during the second stage became the foundation for the gallery walk activity. Gallery Walk is an interactive and collaborative method used to deepen engagement with key themes or concepts identified in the previous stage [33]. Each of the 5 themes from the second stage was assigned to a specific station. These stations served as focal points for discussion and analysis, as shown in Figure 3. Participants worked in pairs, rotating through each station every 15 minutes. At each station, they made notes, posed reflective questions, and provided insights related to the theme. The format was designed to foster dialogue between the pair working together and with previous contributions left by others. This format encouraged deeper engagement and critical analysis. The challenges were identified from this iterative and reflective stage.

### Synthesis of Insights

SP conducted a thematic analysis, a method widely recognized for its flexibility and rigor in qualitative research [34]. The process began with an in-depth familiarization with the data gathered from initial project presentations and the gallery walk. This stage involved careful reading and reflection on the data, allowing for immersion in the presented project materials and the early identification of potential patterns. After familiarizing ourselves with the data, the next step involved generating initial codes. Coding consisted of assigning labels to relevant portions of the data to capture specific aspects of the content. Opportunities in designing connected health systems were primarily identified through the initial project presentations and discussions, while challenges were mainly captured during the gallery walk. Following the coding stage, broader themes were developed by grouping related codes into categories that represented underlying patterns. The iterative structure of the workshop supported a progressive narrowing of focus, ensuring that each phase built upon the previous one.

The final phase of the workshop focused on refining and reaching consensus on the identified themes. After careful revision, these themes were presented and discussed with all workshop participants. This took place during the second half of the workshop day. It provided agreement among participants, thereby addressing the reliability and validity of the analysis.

## Lessons From Practice: Six Connected Health Projects

### Overview

Six projects were presented and discussed in the workshop. They showcased diverse approaches to designing connected health systems.

Project 1 explores remote patient monitoring in Swedish primary care, highlighting its potential to enhance safety for chronic disease management while addressing challenges in patient-provider interaction [35].

Project 2 provides insights into an ongoing project that focuses on improving ICU team shift handovers through EID, emphasizing the need for error reduction and training in managing complex information [24,36].

Project 3 introduces a service process learning cycle to better understand complex health care workflows and foster collaboration among stakeholders [37,38].

Project 4 emphasizes sustainability in health system software, proposing a user-centered co-design framework to address gaps in evaluating sustainability [26,39].

Project 5 presents the Swedish National Medication List, a system aimed at integrating medication data securely across health care providers, with a focus on interoperability and patient safety [30,40].

Finally, Project 6 introduces KokemUX, a user-centered design process to enhance collaboration and streamline design in connected health systems, requiring further refinement for broader applicability [31,41].

Six opportunities were identified when designing in connected health: (1) improving data integration and usability, (2) enhancing collaboration across stakeholders, (3) using a user-centered and iterative design process, (4) addressing complexity in sociotechnical systems, (5) designing for sustainability, and (6) adopting digital infrastructures for seamless communication.

Five challenges were also identified in designing connected health systems: (1) exchange of information, interoperability, and communication; (2) ethical considerations, rules, and regulations; (3) understanding design, evaluation, and standards; (4) actionable data, reliability, quality, and trust in data; and (5) stakeholder involvement.

Opportunities and challenges are thoroughly explored in the following text, one section each.

### Opportunities in Designing for Connected Health

**Figure 4:**
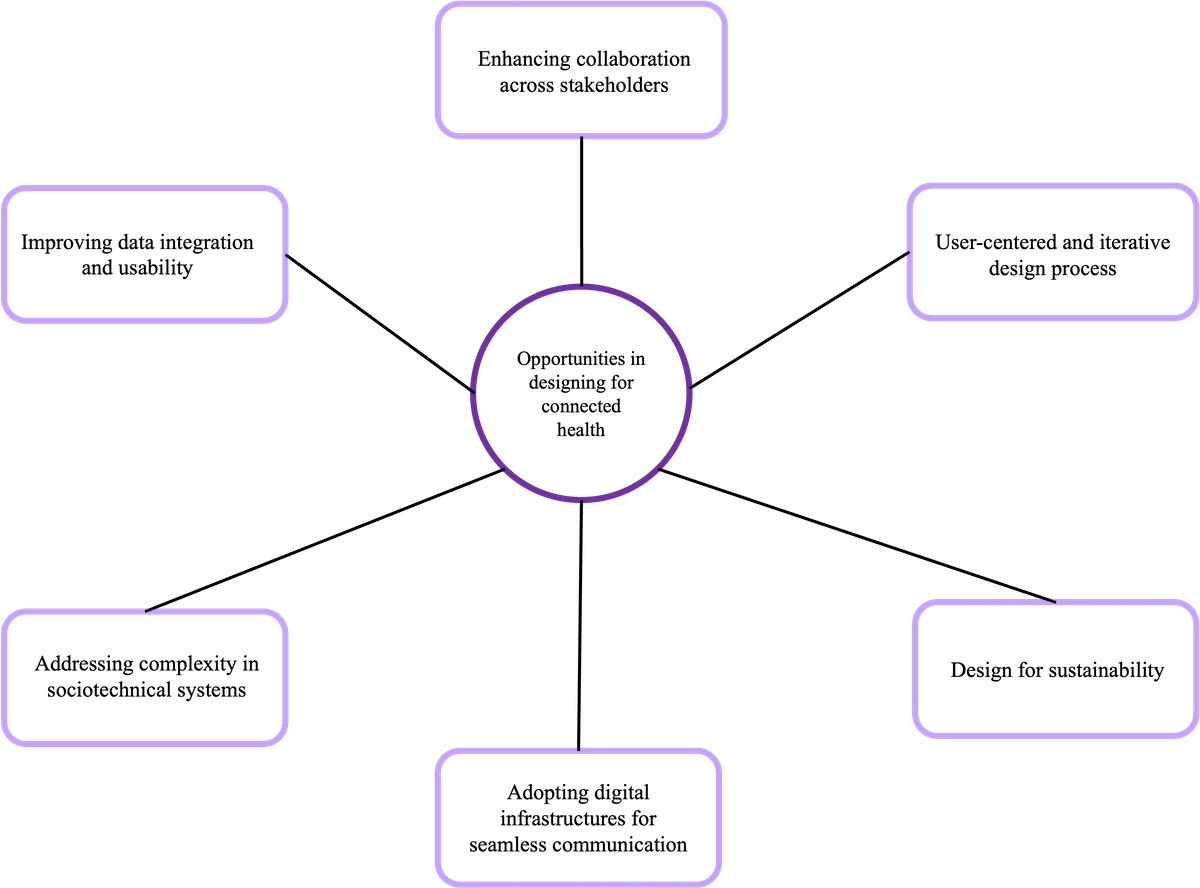
Opportunities in designing for connected health.

This section presents insights from the project presentations and discussions, highlighting 6 themes on opportunities in connected health design. Figure 4 illustrates the opportunities in designing for connected health.

### Theme 1: Improving Data Integration and Usability

With the growing diversity and volume of health care data, including medical records, wearable device outputs, and patient-reported outcomes, designing systems that unify and present this information cohesively is critical. Effective integration ensures that health care providers and patients can easily access, comprehend, and act upon complex data. Approaches such as EID are particularly effective for creating interfaces that align with the cognitive workflows of users. EID supports the visualization of health data in intuitive formats that emphasize actionable insights, enabling health care professionals to make informed decisions swiftly.

### Theme 2: Enhancing Collaboration Across Stakeholders

Collaboration across diverse stakeholders is essential for the success of connected health systems. Health care environments involve numerous actors, including patients, clinicians, IT professionals, designers, and policymakers, each bringing unique perspectives and needs to the table. Aligning these viewpoints requires deliberate efforts to foster shared understanding and coordinated action. Co-design approaches and structured collaboration frameworks, such as Sweden’s exchange contracts for the National Medication List, play a key role in effective teamwork. These frameworks help define shared goals, roles, and standards, bridging communication gaps across institutional and organizational boundaries. Multidisciplinary workshops, stakeholder engagement sessions, and iterative feedback loops are effective strategies for enhancing collaboration. By ensuring that all stakeholders are involved in the design and implementation phases, connected health systems can better address real-world needs, build trust, and increase adoption.

### Theme 3: User-Centered and Iterative Design Processes

Health systems are responsive to real-world needs. These methods emphasize early and frequent engagement with users to refine designs and ensure their relevance and usability. Frameworks such as KokemUX have proven effective in structuring these iterative approaches, allowing developers to incorporate user feedback at every stage of the design cycle. Involving a diverse range of users, including patients with varying levels of digital literacy and health care providers with different technical proficiencies, ensures that the resulting systems are inclusive and practical. Iterative prototyping, usability testing, and scenario-based evaluations allow designers to identify and address issues early, reducing resource wastage and improving system adoption.

### Theme 4: Addressing Complexity in Sociotechnical Systems

Connected health operates within complex sociotechnical systems that encompass interdependent actors, processes, and technologies. Addressing this complexity is a key opportunity in system design. Service process models and ecological design frameworks offer structured methods to understand and manage complex health care systems. These models map critical actors, their interactions, and potential points of failure, enabling designers to create systems that align with real-world workflows and constraints. In patient-centered care, service process models are particularly valuable as they help visualize how patients interact with different services over time, ensuring their needs and experiences remain central. For example, the service process learning cycle supports detailed mapping of health care processes, highlighting inefficiencies and opportunities for improvement [42].

### Theme 5: Design for Sustainability

Sustainability has become an increasingly important consideration in connected health system design. Beyond addressing immediate health care needs, these systems must be designed for long-term use, considering economic, technical, social, and environmental aspects. Co-designing a sustainability framework that integrates user perspectives is a promising approach to ensure that these solutions remain relevant and effective over time. This involves optimizing resource use, such as minimizing energy consumption in digital infrastructures or creating modular systems that can be updated as new technologies emerge. It also considers social impacts such as how the software has an impact on the user and how to keep the product useful over time. Incorporating sustainability assessments into the design process, including evaluations of environmental, social, and economic impacts, ensures that connected health systems align with broader goals of responsible innovation.

### Theme 6: Adopting Digital Infrastructures for Seamless Communication

The adoption of robust digital infrastructures is crucial for enabling seamless communication in connected health systems. Centralized platforms demonstrate the potential for improving coordination and efficiency across health care providers, pharmacies, and patients. These infrastructures facilitate real-time data sharing, ensuring that all stakeholders have access to accurate and up-to-date information. Implementing resource-based architectures, supported by frameworks such as Fast Healthcare Interoperability Resources (FHIR), ensures that systems can scale effectively to handle increasing data volumes. Additionally, these infrastructures provide opportunities for integrating tools such as predictive analytics and decision-support systems, further enhancing their utility. However, the success of digital infrastructures relies on adherence to standards for data security, privacy, and interoperability.

### Challenges in Designing for Connected Health

**Figure 5:**
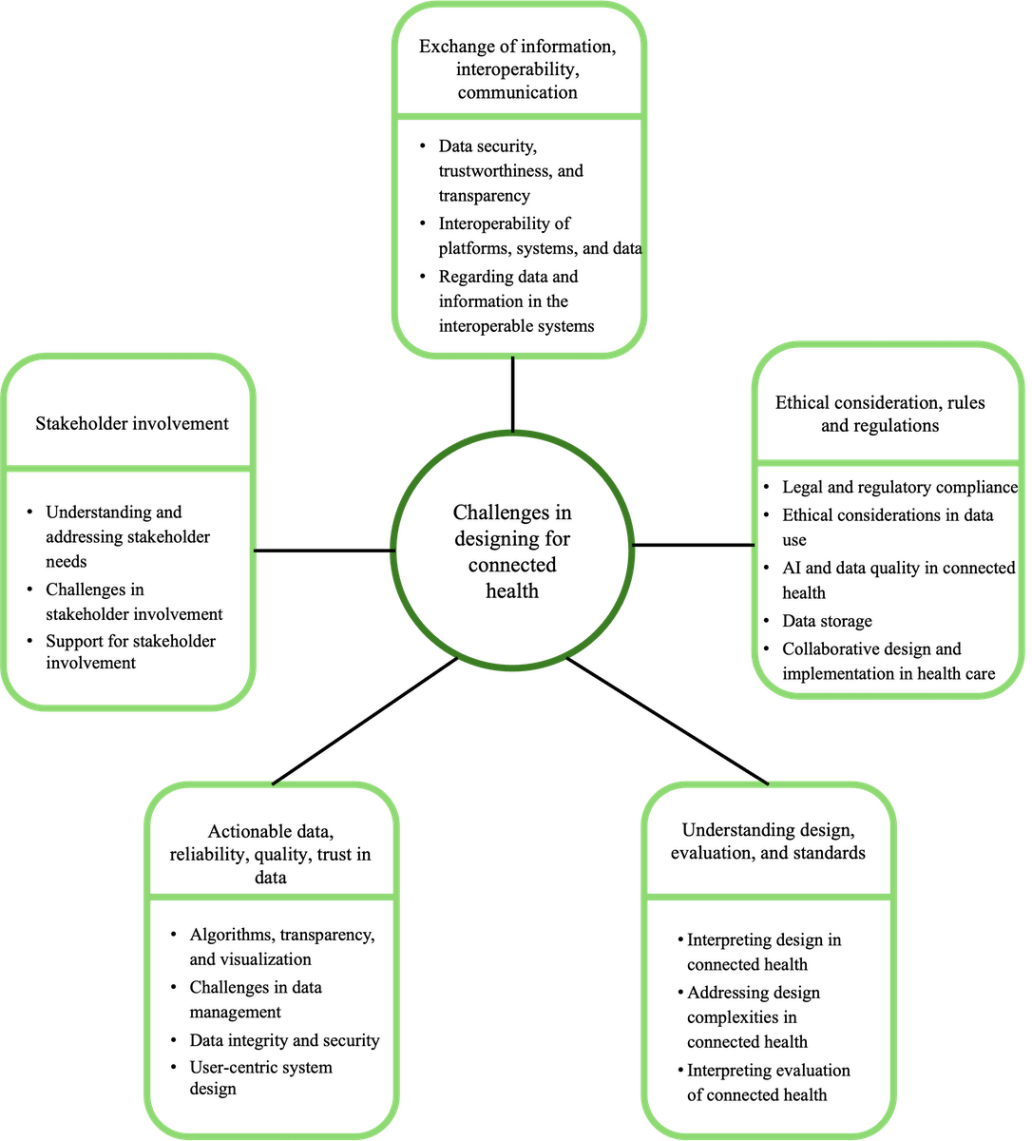
Challenges identified when designing for connected health. AI: artificial intelligence.

This section presents insights from the thematic discussions and gallery walk, where 5 key themes selected from prior discussions were explored in greater depth. These themes represent the core challenges in designing connected health projects. Figure 5 illustrates the challenges identified.

### Challenge 1: Exchange of Information, Interoperability, and Communication

In connected health systems, the seamless exchange of information between various health care institutions is crucial. Interoperability ensures that data collected from different systems can be effectively shared and interpreted. This requires adherence to standards that guarantee the quality and accuracy of data, as well as consideration of legal regulations to ensure compliance and privacy protection.

#### Data Security, Trustworthiness, and Transparency

Data security, trust, and transparency are essential pillars of connected health systems. Maintaining high standards requires secure data practices, compliance with regulations such as GDPR (General Data Protection Regulation) in Europe and HIPAA (Health Insurance Portability and Accountability Act) in the United States, and adherence to standards that ensure consistency and reliability in data exchange. The protection of transmitted data is equally critical and depends on measures such as encryption, integrity checks, secure transmission protocols, and regular audits to safeguard accuracy and prevent breaches.

Equally important is the ethical dimension of transparency. Guided by principles such as “nothing about me without me,” connected health systems must prioritize patient involvement, informed consent, and shared decision-making. Patients should be fully aware of what data is collected, how it is used, who has access to it, and what potential impacts it may have. While encouraging patient engagement is vital, reliance on online sources such as Google or WebMD for self-diagnosis introduces challenges, making it necessary to balance patient empowerment with professional medical expertise.

Clear consent mechanisms and visibility into data flows are central to maintaining trust. Patients must know if their information is processed by artificial intelligence (AI) systems, shared with researchers, or accessed by insurance companies. Transparent communication and accountability ensure that sensitive health information remains secure, ethically managed, and supportive of patient-centered care.

#### Interoperability of Platforms, Systems, and Data

Interoperability of platforms, systems, and data is fundamental to connected health, allowing hospitals, clinics, pharmacies, and laboratories to work together in delivering coordinated patient care. Achieving this requires integrated systems that can share information efficiently, reduce redundancies, and improve outcomes. Interoperability goes beyond transferring raw data; it also involves accurate interpretation and contextualization so that medical information becomes actionable for health care professionals.

Resource-based architectures support this process by managing data, processing power, and bandwidth, while scalable designs ensure systems can handle large data volumes without performance loss. Data exchange must also comply with strict legal, ethical, and technical standards. Mechanisms such as exchange contracts provide validation, accountability, and security, ensuring all parties uphold the quality of shared information. Finally, interoperability should be a priority in system procurement [43], with clear roles, responsibilities, and technical specifications defined to guarantee seamless integration across diverse health care settings.

#### Regarding Data and Information in the Interoperable Systems

In interoperable connected health systems, timely and appropriate responses to data are crucial. Automated alerts, decision-support systems, and predictive analytics help health care professionals act on real-time data efficiently. These systems can be distributed, enhancing privacy and resilience, or centralized, simplifying access but increasing security risks. A key challenge is balancing verbal and digital documentation. Patient interactions span multiple touchpoints, with verbal exchanges often unstructured and inconsistently recorded. Designers must develop methods to effectively capture, process, and integrate both types of data for seamless accessibility and actionability. AI and Internet of Things (IoT)–powered automation further enhance these systems by enabling proactive monitoring, predictive insights, and improved patient outcomes.

### Challenge 2: Ethical Consideration, Rules, and Regulations

Ethical considerations and regulatory compliance are crucial in designing connected health solutions. Designers must address patient privacy, informed consent, and data security while ensuring adherence to regulations such as HIPAA and GDPR to maintain trust and accountability. Ethical design also demands equity and inclusivity, preventing health disparities and ensuring accessibility for diverse populations. By embedding ethics into the design process and staying informed on regulations, stakeholders can create responsible, user-centered solutions that prioritize patient welfare and uphold public trust.

#### Legal and Regulatory Compliance

Legal and regulatory compliance in connected health is complex and often ambiguous, creating challenges in protecting sensitive patient and professional data. Different interpretations of laws can open loopholes that compromise security, making strict adherence to frameworks such as GDPR in Europe and HIPAA in the United States essential. These regulations safeguard privacy, ensure transparency, and uphold patient consent in data exchanges.

The rapid growth of technologies such as AI, wearables, and telemedicine highlights the need for updated legal frameworks that address emerging issues such as data ownership, digital consent, and liability. A lack of specialized knowledge among some legal authorities further complicates oversight, particularly in areas such as AI-driven decision-making and global data flows.

Cross-border data transfer adds another layer of complexity, as differing regional regulations make consistent protections difficult to guarantee. Harmonized international standards and clear global agreements are therefore critical to ensure security, prevent misuse, and build trust in connected health systems worldwide.

#### Ethical Considerations in Data Use

Ethical considerations in data use in connected health go beyond legal compliance and focus on protecting patient rights. Rapid advances in AI and IoT raise concerns about data ownership, consent, and autonomy. While data sharing can foster trust and personalized care, privacy concerns create hesitation, making transparency about collection, use, and protection essential.

Multiple stakeholders often access sensitive health information, and without clear policies on ownership and access, patients risk losing control of their data. The commercialization of health data further undermines trust if used without explicit consent. Patients expect their data to improve care, not be exploited for secondary purposes.

Strong consent processes, limits on commercial use, and clarity around distinctions such as personal versus pseudonymized data are crucial to ethical practice. Ultimately, trust in connected health depends on transparency, respect for autonomy, and clear guidelines that ensure patient data is handled responsibly.

#### AI and Data Quality in Connected Health

The training of AI systems in connected health relies heavily on raw data, necessitating strong anonymization and security measures to protect sensitive personal information. Ensuring data quality and diversity is critical, as biases in training data can lead to biased AI predictions, impacting patient care and trust. While AI training offers significant benefits, such as personalized care, real-time monitoring, and improved decision-making, it also raises concerns about data security, privacy, and potential disparities caused by unrepresentative data. Balanced regulatory responsibility is essential to support innovation while maintaining ethical standards. Current frameworks, such as the EU AI Act, have been criticized for placing excessive compliance burdens on smaller companies, potentially stifling innovation while benefiting larger corporations. Equitably distributing regulatory responsibilities among developers, providers, and end users can foster a more innovative, fair, and ethical connected health ecosystem.

#### Data Storage

Effective data storage in connected health is vital to preserving the integrity, accessibility, and security of sensitive health information. Clear rules on data preservation ensure the accuracy of health records, continuity of care, and support for long-term research, with guidelines on retention, secure storage, and safe disposal, protecting patient privacy. Security in data storage is critical to maintaining patient trust and preventing breaches or cyberattacks, which could compromise safety and confidentiality. Robust security measures and transparent communication about storage practices are essential to reassure patients and safeguard sensitive health data.

#### Collaborative Design and Implementation in Connected Health

Collaborative design and implementation in healthcare underscores the importance of mutual understanding between designers and nondesigners to create effective solutions for connected health. Designers must comprehend the unique challenges and needs faced by health care professionals and patients, while nondesigners need to understand the principles of design thinking and user-centered approaches. This collaborative effort encourages innovation and ensures the resulting products are practical, user-friendly, and tailored to actual needs. Through open communication and iterative feedback, the gap between technical design and real-world application can be bridged, enhancing health care solutions’ overall quality. Implementing a connected health system can significantly benefit various stakeholders, including patients who gain personalized care and real-time monitoring and health care providers who can streamline workflows and make more informed decisions. Health organizations may also benefit from improved efficiency and cost savings. However, disparities exist; vulnerable populations without access to technology or digital literacy skills may face barriers, while health care professionals resistant to change or lacking training might need help to adapt. Addressing these inequities is crucial to ensure equitable access and maximize the system’s positive impact.

### Challenge 3: Understanding Design, Evaluation, and Standards

Design and evaluation standards are crucial in connected health as they ensure consistency, quality, and safety in health solutions. By providing a framework for creating reliable and effective products, these standards help maintain regulatory compliance and protect patient privacy, fostering trust in health care technologies. They facilitate interoperability between different systems, enhancing the overall user experience through an intuitive and accessible design. Furthermore, evaluation standards support continuous improvement.

#### Interpreting Design in Connected Health

Design is a powerful tool in connected health, helping to solve complex problems and create new opportunities. By applying user-centered principles, designers can address challenges faced by patients and providers while fostering innovation that improves accessibility, delivery, and outcomes. Design is not only a process but also a mindset—one that values creativity, empathy, and interdisciplinary collaboration to continuously refine ideas and solutions.

At its core, design shapes user interaction and satisfaction. Effective communication and interaction design ensure that solutions are intuitive, functional, and aligned with user needs. Asking “why” clarifies purpose, while asking “how” guides implementation, grounding design in real-world contexts and practical strategies.

Design is also a continuous learning process. Iterative feedback, creativity within organizational constraints, and adherence to standards and best practices support quality, safety, and scalability. A life cycle approach further ensures adaptability, allowing connected health systems to evolve with technological advances and user feedback. This adaptability is essential for sustaining relevance, resilience, and trust, ultimately leading to better health outcomes and user satisfaction.

#### Addressing Design Complexities in Connected Health

Developing effective connected health solutions faces several challenges. Overreliance on specific design methodologies can limit innovation and neglect broader user needs, making solutions less adaptable. Cross-disciplinary collaboration is therefore crucial for generating more comprehensive designs that address diverse stakeholder perspectives. The fast-changing landscape of connected health—driven by new technologies, evolving regulations, and shifting patient expectations—adds complexity, requiring multidisciplinary approaches and scalable solutions. Short design life cycles further constrain evaluation and refinement, often producing systems that fail to meet real-world demands. Extending these cycles to incorporate user feedback helps ensure designs remain user-centered and effective.

Designers must also compare and combine different approaches, such as user-centered and agile methods, to build hybrid strategies that balance strengths and limitations. Flexibility is key, as solutions must adapt to technological change, health care needs, and user input. At the same time, connected health must confront “wicked problems” such as health inequities and chronic disease management, which demand collaborative, incremental strategies rather than definitive solutions. Finally, sustained user engagement is vital: intuitive design, personalization, clear communication, and ongoing support are necessary to retain users and achieve meaningful health outcomes.

#### Interpreting Evaluation of Connected Health

Interpreting evaluation of connected health requires assessing both the outcomes of solutions and the methods behind their creation. Evaluating “the solution” measures impact through patient satisfaction, clinical efficacy, usability, and efficiency, while evaluating “the way” examines design processes, collaboration, and adaptability. Combining qualitative and quantitative methods—such as surveys, interviews, and usability testing—offers a comprehensive view and supports continuous improvement. By systematically analyzing this data within an “experiences factory,” stakeholders can identify trends and patterns, enabling iterative improvements that enhance usability and effectiveness.

Compliance with regulations such as GDPR or HIPAA is essential, but focusing only on legal standards risks overlooking user experience. A balanced approach that integrates compliance with patient and provider feedback ensures solutions are both safe and meaningful. Metrics such as engagement rates, retention, and qualitative user insights help refine systems iteratively. Life cycle assessment adds another layer, considering environmental, social, and economic impacts to promote sustainability. Together, these strategies create connected health solutions that are effective, user-centered, and adaptable over time.

### Challenge 4: Actionable Data, Reliability, Quality, and Trust in Data

This theme focuses on deriving clinically useful insights that hold tangible relevance for health care providers and patients. For data to be actionable, systems must consistently produce accurate and repeatable results, fostering trust in their reliability. Building this trust requires robust validation processes, transparent methodologies, and evidence of efficacy. By prioritizing these principles, connected health solutions can ensure data quality while empowering stakeholders with meaningful and dependable information.

#### Algorithms, Transparency, and Visualization

In connected health, algorithms using machine learning and AI are indispensable for processing vast health data, identifying patterns, and predicting outcomes. However, their potential is tied to addressing biases, inaccuracies, and unintended outcomes that can impact clinical trust and outcomes. Transparency in algorithmic processes is essential to instill confidence among health care providers and patients, allowing stakeholders to review how data is processed and decisions are reached. Clear visualizations further enhance usability, enabling health care professionals to interpret complex data effectively through intuitive displays. Backup systems bolster reliability, ensuring uninterrupted data availability during failures. Additionally, creating clear rules for algorithmic decision-making—with input from clinicians and patients—improves system accountability. Access to raw data and methodologies enables stakeholders to understand and validate conclusions, reinforcing trust in connected health systems.

#### Challenges in Data Management

Connected health systems face numerous challenges in managing the ever-growing influx of data. The sheer volume can overwhelm users, making prioritization mechanisms and intelligent filtering vital to reduce noise and maintain focus on actionable insights. Biases in algorithms further complicate data management, as these systems must ensure equitable representation across diverse populations to prevent health care disparities. The quality and variability of data remain persistent challenges, as not all sources are equally reliable; robust validation processes are essential to maintain data legitimacy. For data to be impactful, it must also be presented in user-friendly formats and contextualized with timelines and historical relevance. Tackling these challenges head-on ensures that data in connected health systems remains actionable, equitable, and effective in improving patient care.

#### Data Integrity and Security

Data integrity and security are foundational to building trust and reliability in connected health. Accuracy begins at the source, necessitating high-quality sensors and rigorous validation processes. Systems must prioritize clinically significant data to reduce clutter and deliver insights that truly matter to health care professionals and patients. Monitoring the quality of data throughout the chain, from input to output, minimizes errors and ensures consistent reliability. Data security is equally critical; unauthorized readings or tampering pose significant risks to patient safety and privacy. Using robust encryption, authentication, and monitoring mechanisms safeguards sensitive health information. Additionally, refining algorithms to reduce false positives and negatives mitigates the risk of misdiagnoses, ensuring data are not only accurate but also actionable in the context of patient care.

#### User-Centric System Design

A connected health system must be designed with the end user in mind, ensuring a balance between functionality and simplicity. Overloading health care providers with frequent and noncritical alerts can lead to alarm fatigue, desensitizing them to critical warnings. Smarter alert systems that prioritize and contextualize notifications improve response rates and reduce frustration. Similarly, finding the optimal frequency for data collection avoids both gaps and overload, tailoring intervals to clinical requirements for maximum efficiency. Continuous feedback mechanisms between users and systems enable ongoing refinement, correcting errors and improving usability over time. By placing health care providers and patients at the center of design, connected health systems can enhance decision-making and outcomes while reducing cognitive and operational burdens.

### Challenge 5: Stakeholder Involvement

Stakeholder involvement is a critical aspect of designing connected health solutions, ensuring that the needs, preferences, and challenges of all parties are adequately addressed. This includes patients, caregivers, health care professionals, designers, IT professionals, and policymakers. Meaningful engagement fosters collaboration, builds trust, and creates solutions that are both user-centered and sustainable. Effective stakeholder involvement involves a variety of methods, such as focus groups, interviews, and workshops, to gather insights and cocreate solutions.

#### Understanding and Addressing Stakeholder Needs

Effective stakeholder engagement is crucial for the design, implementation, and evaluation of connected health solutions. Qualitative methods such as focus groups and interviews provide structured insights into stakeholder needs, ensuring designs are grounded in real-world contexts. Continuous engagement throughout the design life cycle enables iterative improvements, allowing solutions to evolve based on feedback and changing requirements. Identifying key stakeholders for long-term impact ensures sustainability, scalability, and alignment with evolving health care demands. A holistic understanding of stakeholder needs helps address the diverse requirements of patients, caregivers, and health care professionals. Structured engagement strategies prevent redundant input requests, focusing efforts on actionable insights to save time and resources. Involving users—such as clinicians and tech-savvy patients—ensures practical, relevant, and expertise-driven designs. Stakeholders must fully understand the solution’s purpose and impact to provide meaningful contributions. Building trust and demonstrating value fosters stakeholder ownership, leading to stronger commitment and long-term success. By addressing these aspects, connected health solutions can achieve greater relevance, usability, and impact in health care.

#### Challenges in Stakeholder Involvement

Effectively engaging stakeholders in connected health design requires addressing key challenges. Recruitment in health care is time-intensive and must ensure diverse, representative participation while adhering to ethical standards. Power hierarchies—between doctors and patients or senior and junior staff—can marginalize voices, leading to biased design inputs. Balancing stakeholder influence across disciplines prevents dominance by any single group and promotes equitable, collaborative decision-making. Addressing unconscious biases, such as elementary bias, is essential to ensuring fair design processes that reflect diverse perspectives. Sustaining stakeholder involvement is another challenge, as competing priorities may lead to disengagement. Retention strategies should emphasize participants’ value, fostering ongoing commitment. Overcoming these challenges enhances stakeholder engagement, making connected health solutions more inclusive, effective, and impactful.

#### Support for Stakeholder Involvement

It is crucial to foster effective collaboration among diverse stakeholders in connected health. Establishing a shared terminology and clear communication channels is crucial to bridging gaps between individuals with different expertise and ensuring smooth and productive interactions. The involvement of interdisciplinary groups, including professionals from health care, technology, and design, encourages innovation by integrating multiple perspectives and fostering holistic solutions. Additionally, an education support plan is essential for equipping stakeholders—especially end users—with the knowledge and resources they need to understand and adapt to connected health technologies. By prioritizing these elements, stakeholder collaboration becomes more cohesive, inclusive, and effective, driving the success of connected health initiatives.

## Reflections and Recommendations

### Overview

This paper presents key opportunities and challenges in connected health, categorized based on project discussions and gallery walk insights. Six opportunities emerged for advancing design through diverse methodologies: (1) improving data integration and usability, (2) enhancing collaboration across stakeholders, (3) using a user-centered and iterative design process, (4) addressing complexity in sociotechnical systems, (5) designing for sustainability, and (6) adopting digital infrastructures for seamless communication. These approaches effectively addressed user engagement, workflow integration, and system interoperability, enhancing patient and provider experiences by aligning health care technologies with real-world needs.

Insights from the gallery walk revealed five major challenges in designing connected health systems: (1) exchange of information, interoperability, and communication; (2) ethical consideration, rules, and regulations; (3) understanding design, evaluation, and standards; (4) actionable data, reliability, quality, and trust in data; and (5) stakeholder involvement.

### Opportunities in Designing for Connected Health

The results indicated that improving data integration and usability is a critical opportunity for advancing connected health systems. The complexity of health care data often poses challenges for both patients and providers, limiting its accessibility and actionable potential. This emphasis on usability can foster significant benefits, including improved communication between health care providers and patients, leading to more personalized care. However, achieving these outcomes requires addressing several challenges. Designers must account for the diverse needs and varying technical proficiencies of end users, including patients with limited digital literacy [44,45]. Ensuring data security and compliance with regulatory frameworks, such as GDPR and HIPAA, remains essential to building trust in these systems [46]. Future work could explore how usability-focused design methods can be systematically integrated into the development lifecycle of connected health systems to maximize their impact.

Collaboration among diverse stakeholders is a fundamental aspect of designing and implementing effective connected health systems. Given the complexity of health care ecosystems, where multiple actors—including patients, clinicians, IT professionals, designers, and policymakers—contribute to service delivery and system development, achieving alignment among these groups is critical. The findings suggest that connected health systems that prioritize participatory approaches are more likely to meet the diverse needs of their users, leading to more sustainable and effective digital health interventions. Despite the clear benefits, ensuring effective collaboration across stakeholders presents ongoing challenges [47]. Power imbalances, differences in technical knowledge, and organizational silos can impede open communication and shared decision-making [48,49]. Additionally, stakeholder engagement requires sustained effort, as participation levels may fluctuate due to competing responsibilities or resource constraints. Addressing these barriers necessitates the development of transparent communication channels, clear governance structures, and mechanisms for continuous involvement throughout the design and implementation process. Future research could explore how emerging digital tools, such as AI-driven decision support and data-sharing platforms, can further streamline collaboration in connected health systems [50].

The findings emphasize the importance of user-centered and iterative design approaches in developing connected health systems that are responsive to real-world needs. Several studies also highlight that engaging users early and frequently in the design process ensures that digital health solutions remain relevant, usable, and effective in diverse health care environments [51]. This can minimize resource wastage, enhance user experience, and influence adoption rates by delivering solutions that align with stakeholder expectations and practical health care workflows [52]. Another key advantage of iterative design is its ability to enhance inclusivity [53]. By involving a diverse range of users—patients, caregivers, clinicians, and IT professionals—the design process accounts for different needs and contexts. This ensures that connected health systems do not inadvertently exclude certain populations due to accessibility barriers or overly complex interfaces. Despite its advantages, implementing user-centered and iterative design processes comes with challenges. These include the time-intensive nature of repeated iterations, the potential for conflicting user feedback, and the need for adequate resources to conduct usability testing [54]. Future research should explore how emerging technologies, such as AI and predictive analytics, can further optimize user feedback integration, making the iterative process more efficient and scalable.

Our findings also highlight the intricate nature of connected health systems, which operate within sociotechnical ecosystems composed of diverse actors, interdependent processes, and evolving technologies. Effectively addressing this complexity presents a significant opportunity in system design, ensuring that digital health solutions remain adaptable, scalable, and aligned with real-world health care needs. Given the dynamic and often fragmented nature of health care environments, structured approaches are necessary to manage these complexities systematically [49]. However, interdisciplinary collaboration is also essential, as addressing sociotechnical complexity requires input from health care professionals, IT specialists, policymakers, and end users [55]. Future research could explore how emerging technologies such as AI and predictive analytics can enhance the modeling and management of these complex systems, making connected health solutions more adaptive and resilient.

Our results echo the increasing emphasis on sustainability in connected health system design, reflecting a broader shift in digital health innovation toward long-term usability, adaptability, and reduced environmental impact. Sustainability in eHealth design encompasses technological, economic, social, and environmental factors [56,57]. Key challenges include escalating health care costs, aging populations, and increasing chronic diseases [58]. To address these issues, eHealth solutions must be developed through participatory processes, focusing on value creation and user needs [59]. A holistic approach involving ethical design, eco-audits, and effective policies is necessary for sustainable eHealth systems [60]. Assessment frameworks, such as the one proposed by [57], can help evaluate the sustainability of eHealth solutions, addressing critical issues in feasibility and long-term viability.

Robust digital infrastructures are increasingly recognized in scholarly literature as a cornerstone of effective connected health systems. Research highlights the significance of structured digital platforms in improving coordination among health care providers, pharmacies, patients, and caregivers [55,61]. Scalability is a key concern in digital health infrastructure, particularly with the growing volume of health data [62]. Research suggests that resource-based architectures, such as those supported by FHIR, provide the necessary flexibility and scalability to accommodate expanding health care needs [63]. Several studies emphasize that predictive analytics can leverage real-time health data to forecast patient deterioration, optimize resource allocation, and enhance clinical decision-making [64]. Additionally, integrating AI-driven decision-support systems into digital infrastructures significantly improves diagnostic accuracy and treatment outcomes by providing clinicians with data-driven insights.

While the workshop primarily focused on design opportunities and challenges, it is important to acknowledge that the successful implementation of connected health solutions also depends on economic and organizational feasibility. Business models, funding strategies, and resource allocation directly shape whether innovative designs can move beyond prototypes into sustainable practice [65]. Issues such as reimbursement models, procurement processes, and organizational readiness often determine adoption in health care settings [66]. Long-term scalability further requires alignment with institutional priorities and financial sustainability, ensuring that solutions remain viable beyond initial project phases [67]. Although these aspects were not the central focus of the workshop, future research and cross-sector collaborations should explicitly integrate economic and organizational perspectives to strengthen the practical utility of design recommendations.

### Challenges in Designing for Connected Health

The results emphasize the critical role of seamless data exchange and interoperability in connected health systems. Recent studies have supported this and argue that achieving interoperability requires adherence to shared communication protocols and data models to avoid fragmented care [68]. However, achieving interoperability remains challenging due to diverse and heterogeneous information and communications technology (ICT) tools, methods, and proprietary models used in health care organizations [69]. Key enablers of interoperability include health information exchange, interoperability standards, and application programming interfaces [68]. Standardization of health care terminology, education strategies, and addressing privacy and security concerns are essential for achieving complete interoperability [69,70]. Recent advancements, such as the CARE CONNECT framework, show promise in improving health information exchange and interoperability across various health care platforms [71]. Despite progress, challenges persist, including privacy concerns, organizational barriers, and technical limitations, necessitating ongoing research and development in this field [69,70]. Similarly, Brokel et al [72] emphasize the necessity of aligning institutional workflows with technical standards to enable the standardization of care processes and facilitating quality improvement initiatives. Data security and compliance frameworks such as GDPR and HIPAA are foundational for trust in connected health. Implementing these regulations in IoT health care infrastructures remains challenging, with proposed solutions including edge computing and encryption techniques [73]. Blockchain technology has emerged as a promising solution for managing electronic health records while complying with GDPR and HIPAA requirements [74]. To address the challenges of sharing health big data, a semantically rich Compliance Enforcement Framework has been developed, incorporating trust scores and ontologies to ensure regulatory compliance in real-time data exchange scenarios [75]. Transparency in data use and patient engagement, such as the principle “nothing about me without me,” has been emphasized in recent studies such as Montague et al [76].

Our results also highlight the need for ethical considerations in data governance, particularly with advancements in AI and IoT. Key challenges include protecting privacy while using personal data for societal benefits [77]. Data ecosystems enabling the use and reuse of big datasets raise ethical questions around privacy, accountability, ownership, accessibility, and motivation [78]. Effective governance must address not only privacy and security but also unexpected outcomes such as clinician deskilling, algorithmic bias, and equitable access to AI benefits [79]. The complexity of networked entities in data governance necessitates new approaches beyond traditional policy models. Balancing innovation with ethical considerations is crucial for protecting patient rights and maintaining trust in connected health systems [80]. Ongoing research and stakeholder engagement are essential to evolve ethical standards in line with technological advancements in connected health. Studies emphasize the need for critical appraisal of data and algorithms to address potential biases in connected health systems [81]. The validity of data and inferences drawn from AI systems are likely to be biased, regardless of sample size [81]. Studies have stressed the importance of raising awareness about algorithmic decision-making in connected health and its impact on care delivery [82]. To address these issues, experts call for open science approaches to mitigate bias in big data and AI for health care [83]. Additionally, building trustworthy and explainable AI in connected health systems remains challenging, with low methodological quality and high bias risk being major concerns [84].

Our results also show the need to understand design, evaluation, and standards in connected health. A focus on user-centered, iterative design and evaluation that involves end users is crucial throughout the design process. Studies have suggested a 3-phase methodology that includes creating use cases with user feedback, expert usability inspections, and user testing with target end users [12]. Recent studies have also highlighted the relevance of activity theory to provide a theoretical framework for UCD in connected health, offering a broader contextual analysis for iterative design and evaluation [85]. UCD methods have proven valuable in addressing barriers to the diffusion of smart and connected health applications, highlighting the importance of user involvement from the early stages of development [86]. Implementing UCD approaches in connected health projects can lead to improved usability, human factors, and user experience, ultimately increasing the likelihood of achieving intended health outcomes [12,87,88].

Actionable and reliable data are central in connected health to improving patient outcomes as supported by our results. Recent studies suggest that the digitization of medical records and the increasing transparency of health care data are transforming the industry, driving innovation and creating significant value [89]. This abundance of complex health systems data presents opportunities for collaboration between health care professionals, informaticians, and researchers to transform data into actionable information for improving clinical outcomes [90]. However, challenges exist in collecting reliable data for evaluating digital health care applications. Recent studies suggest a participant-driven data collection platform, incorporating interpretable data preparation, and systematic storage [91]. The concept of actionable data in health care varies depending on the context, such as exploratory research or clinical diagnostics. Evaluating the trustworthiness and actionability of data, for instance, depends on the goals and resources within the specific situation of inquiry and the social epistemology of standards [92].

Stakeholder engagement is crucial in connected health as suggested in our results [93]. Effective engagement involves identifying stakeholders, understanding their interests and power dynamics, and developing strategies for integration [93,94]. Prior research suggests a multilevel approach to engagement can overcome barriers and ensure meaningful participation in governance, network design, and implementation [95]. The degree of connectivity between new health ICT product-service systems and their operating environment impacts stakeholder engagement decisions in early-stage development [96]. Evaluating connected health solutions requires a holistic framework that considers multiple stakeholder perspectives, focusing on end user perception, business growth, quality management, and health care practice [94]. By involving stakeholders at various levels, from research team members to survey respondents, organizations can develop patient-centered research programs and increase the relevance of their work to broader audiences [95].

Based on the results, we present our recommendations for designing in connected health in Table 2.

**Table 2:** Recommendations for designing in connected health.

Recommendations	Description
Prioritize interoperability through standardization	Adopt global standards and frameworks (eg, HL7 FHIR^a^) to ensure seamless data exchange across platforms, institutions, and countries. Focus on creating unified exchange contracts that define technical, ethical, and legal parameters, addressing inconsistencies in health care data sharing and interoperability.
Design for scalability and adaptability	Build modular systems with resource-based architectures to accommodate growing data volumes and technological advancements. Ensure solutions are flexible enough to integrate new technologies such as AI^b^, IoT^c^, and predictive analytics while maintaining performance and usability.
Enhance stakeholder engagement through co-design	Actively involve diverse stakeholders, including patients, health care providers, and policymakers, in co-design processes to address their unique needs. Use structured methods such as focus groups and workshops to foster collaboration, reduce power hierarchies, and align on shared goals.
Embed ethical and legal safeguards in system design	Develop clear consent mechanisms, anonymization protocols, and data ownership policies to enhance transparency and trust. Integrate ethics as a core component of the design process to address privacy concerns, data security, and equitable access for vulnerable populations.
Leverage user-centered and iterative design processes	Use frameworks such as KokemUX and Service Process Learning Cycles to ensure user involvement at all stages of development. Iterate designs based on real-world feedback to create solutions that are intuitive, efficient, and tailored to specific health care workflows.
Implement robust data management and security measures	Ensure data integrity by integrating high-quality sensors, encryption protocols, and real-time monitoring systems. Develop systems that prioritize critical alerts, reduce false positives/negatives, and provide actionable insights to minimize alarm fatigue for health care providers.
Incorporate sustainability as a core design principle	Design systems with long-term usability and minimal environmental impact in mind. Develop frameworks to evaluate and optimize energy efficiency, data storage practices, and system resilience to ensure sustainable connected health solutions.

^a^FHIR: Fast Healthcare Interoperability Resources.

^b^AI: artificial intelligence.

^c^IoT: Internet of Things.

### Looking Ahead: Future Directions for Connected Health Design

The paper presents valuable insights into designing connected health systems, but certain limitations highlight areas for future research. The findings were derived from 6 projects, which, while diverse in methodology and context, may not fully capture the breadth of challenges and opportunities across the field. Expanding the scope to include more projects and varied contexts would provide a richer understanding of the design landscape in connected health. The participant group, though multidisciplinary, may have underrepresented key stakeholders such as patients, regulatory bodies, or underresourced health care providers, limiting the diversity of perspectives. Future workshops and studies should prioritize more inclusive engagement to ensure that all critical voices are heard. Many of the insights were context-specific, reflecting health care settings and geographic regions. Research is needed to explore how these findings apply in different regulatory environments, health care systems, and cultural contexts to ensure global applicability. The workshop also emphasized design and technical aspects, with less focus on economic and organizational models essential for sustaining and scaling connected health systems. Future research should integrate these dimensions, examining viable business models and strategies for long-term implementation. In addition, this paper is primarily technical and methodological in focus, with less emphasis on user engagement and patient-centered care. Instead, the paper contributes by complementing patient-centered discussions with insights into design methodologies in connected health. Finally, the limited duration of the workshop constrained the depth of exploration and validation of the identified themes and challenges. Longer engagements or longitudinal studies could provide more robust and actionable insights, enabling continuous refinement and adaptation of connected health design methodologies.

## Conclusions

Designing connected health systems presents a complex interplay of opportunities and challenges. Insights gathered from the workshop highlight the transformative potential of these systems to enhance health care through sustainable and collaborative approaches. Leveraging innovative design methodologies such as UCD, service design, EID, and KokemUX in connected health projects can address critical areas such as usability, interoperability, and patient engagement. However, significant barriers persist, including regulatory compliance, data security, and equitable stakeholder involvement. Overcoming these requires a multipronged strategy. Iterative, user-focused design methodologies must be coupled with robust legal frameworks to ensure compliance and build trust. Collaborative stakeholder engagement, interdisciplinary approaches, and clear communication are essential to fostering innovation and aligning diverse needs. Furthermore, systems must be adaptable, incorporating continuous feedback to evolve with technological advancements and shifting health care demands. The findings emphasize that the success of connected health lies in balancing technical innovation with ethical considerations and user priorities. Future research and practice should focus on refining design processes, fostering inclusivity, and addressing the scalability and sustainability of solutions. By doing so, connected health can fulfill its promise of delivering impactful, equitable, and efficient health care outcomes.

## Abbreviations

AI: artificial intelligence
EID: ecological interface design
FHIR: Fast Healthcare Interoperability Resources
GDPR: General Data Protection Regulation
HIPAA: Health Insurance Portability and Accountability Act
ICT: information and communications technology
ICU: intensive care unit
IoT: Internet of Things
UCD: user-centered design

## Data Availability

All data generated or analyzed during this study are included in this published article.
